# Investigations of barley stripe mosaic virus as a gene silencing vector in barley roots and in *Brachypodium distachyon *and oat

**DOI:** 10.1186/1746-4811-6-26

**Published:** 2010-11-30

**Authors:** Andrzej Pacak, Katrin Geisler, Bodil Jørgensen, Maria Barciszewska-Pacak, Lena Nilsson, Tom Hamborg Nielsen, Elisabeth Johansen, Mette Grønlund, Iver Jakobsen, Merete Albrechtsen

**Affiliations:** 1 Department of Genetics and Biotechnology, Faculty of Agricultural Sciences, Aarhus University, Thorvaldsensvej 40, 1871 Frederiksberg C, Denmark; 2 Department of Plant Biology and Biotechnology, Faculty of Life Sciences, University of Copenhagen, Denmark; 3 Biosystems Division, Risø National Laboratory for Sustainable Energy, Technical University of Denmark, PO Box 49, DK-4000 Roskilde, Denmark; 4 Current Address: Department of Gene Expression, Adam Mickiewicz University, Umultowska 89, 61-614 Poznan, Poland; 5 Current Address: Department of Agriculture and Ecology, Faculty of Life Sciences, University of Copenhagen, Thorvaldsensvej 40, 1871 Frederiksberg C, Denmark; 6 Current Address: Department of Forest Genetics and Plant Physiology, Swedish University of Agricultural Sciences, S901-83 Umeå, Sweden; 7 Current Address: Section for Sustainable Biotechnology, Department of Biotechnology, Chemistry and Environmental Engineering, Copenhagen Institute of Technology, Aalborg University, Ballerup, Denmark

## Abstract

**Background:**

Gene silencing vectors based on *Barley stripe mosaic virus *(BSMV) are used extensively in cereals to study gene function, but nearly all studies have been limited to genes expressed in leaves of barley and wheat. However since many important aspects of plant biology are based on root-expressed genes we wanted to explore the potential of BSMV for silencing genes in root tissues. Furthermore, the newly completed genome sequence of the emerging cereal model species *Brachypodium distachyon *as well as the increasing amount of EST sequence information available for oat (*Avena *species) have created a need for tools to study gene function in these species.

**Results:**

Here we demonstrate the successful BSMV-mediated virus induced gene silencing (VIGS) of three different genes in barley roots, i.e. the barley homologues of the *IPS1*, *PHR1*, and *PHO2 *genes known to participate in Pi uptake and reallocation in Arabidopsis. Attempts to silence two other genes, the Pi transporter gene *HvPht1;1 *and the endo-β-1,4-glucanase gene *HvCel1*, in barley roots were unsuccessful, probably due to instability of the plant gene inserts in the viral vector. In *B. distachyon *leaves, significant silencing of the *PHYTOENE DESATURASE *(*BdPDS) *gene was obtained as shown by photobleaching as well as quantitative RT-PCR analysis. On the other hand, only very limited silencing of the oat *AsPDS *gene was observed in both hexaploid (*A. sativa*) and diploid (*A. strigosa*) oat. Finally, two modifications of the BSMV vector are presented, allowing ligation-free cloning of DNA fragments into the BSMV-γ component.

**Conclusions:**

Our results show that BSMV can be used as a vector for gene silencing in barley roots and in *B. distachyon *leaves and possibly roots, opening up possibilities for using VIGS to study cereal root biology and to exploit the wealth of genome information in the new cereal model plant *B. distachyon*. On the other hand, the silencing induced by BSMV in oat seemed too weak to be of practical use. The new BSMV vectors modified for ligation-free cloning will allow rapid insertion of plant gene fragments for future experiments.

## Background

*Barley stripe mosaic virus *(BSMV) is a single-stranded RNA virus with three genome components termed α, β, and γ [1]. Infectious clones of several strains of BSMV have been constructed [2]. To initiate infection, *in vitro *transcripts of all three genome components are mixed and rubbed onto leaves of host plants. Vectors based on BSMV have been shown capable of inducing efficient gene silencing in leaves of barley (*Hordeum vulgare*) [3] and wheat (*Triticum vulgare*) [4]. Subsequently, numerous studies have used BSMV vectors for gene silencing studies in barley or wheat (reviewed in [1,5]). However, only a few studies have explored the capacity of BSMV for gene silencing in other species (in *Haynaldia villosa*, [6], and *Brachypodium distachyon *ecotype ABR-1, [7]). In addition to BSMV, two vectors based on *Brome mosaic virus *have been reported to induce gene silencing in barley, maize and rice [8,9], but these vectors have not yet been widely used. Furthermore, no reports have appeared of virus induced gene silencing in monocot roots. Genes expressed in roots are involved in important processes such as root development, nutrient uptake, and pathogen resistance. Therefore, tools for studying root expressed genes are needed. Due to our interest in phosphorous (P) starvation responses, we have chosen some important genes of interest for phosphate (Pi) acquisition and the P-starvation response for the exploration of the usefulness of VIGS in barley roots. Previously, virus induced gene silencing (VIGS) has been successfully demonstrated in roots of several dicot plant species using vectors based on *Tobacco rattle virus *[10], *Pea early browning virus *[11,12], *Tomato yellow leaf curl China virus *[13], or *Bean pod mottle virus *[14].

*B. distachyon *is a small, fast-growing, self fertile member of the grass subfamily Pooideae, which also encompasses the major cereals wheat and barley as well as forage grasses. *B. distachyon *is being implemented as a model species for grasses and cereals due to several attractive features including a relative small (~ 275 Mb) genome [15]. In recognition of the importance of developing a model grass, the US DoE Joint Genome institute has funded the sequencing of the *B. distachyon *genome and the sequence was published in 2010 [16]. Although genetic transformation protocols for *B. distachyon *have been established [15,17-19], the VIGS technique would be a useful supplement as a faster and simple means of exploring gene function.

Oat (Avena) is another member of the Poaceae family. Most cultivated oat varieties belong to the hexaploid *A. sativa *with an estimated 1C genome size of 13000 Mbp but diploid wild oat and tetraploid varieties exist. Although the large oat genome remains relatively unexplored compared to wheat, barley, and indeed *B. distachyon*, genetic maps of oat are being developed [20,21]. Furthermore large oat EST sequencing programmes are in progress, and already 16k ESTs of diploid *A. strigosa *root material and around 25k *A. sativa *and 54k *A. barbata *ESTs are publicly available ([22,23] and unpublished GenBank accessions). Hexaploid oat cultivars (*A. sativa*) have been successfully transformed using biolistic [24] and *Agrobacterium*-mediated [25] transformation techniques, but these techniques are time consuming. Thus a VIGS approach for oat could greatly reduce the time and effort needed to identify and study gene function in oat.

Here we show that BSMV can be used to induce VIGS in roots of barley, although attention to the stability of the inserts in the vector is recommended. Furthermore, successful VIGS in leaves of *B. distachyon *was obtained. On the other hand, the silencing efficiency in *Avena *species appeared too low to be of practical value. Finally, two BSMV vectors adapted for ligation-free insertion of gene fragments are presented.

## Results

### VIGS in barley roots

**Figure 1:**
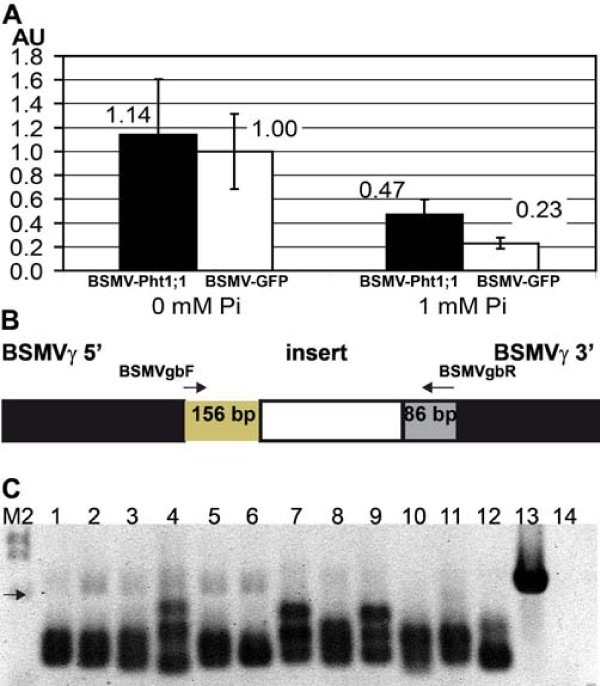
**Attempt at silencing *HvPht1;1 *expression in barley roots**. **A ***HvPht1;1 *mRNA expression levels in root tissue determined by qRT-PCR (normalization to ubiquitin). Plants were inoculated with either BSMV-Pht1;1 (black bars) or BSMV-GFP^375 ^(white bars). Plants were grown in hydroponic culture containing 0 or 1 mM Pi. Each bar represents three samples, error bars denote standard deviations. AU - Arbitrary units. **B **Schematic view of BSMVγ with introduced insert. Arrows show the position of the primers BSMVgbF and BSMVgbR used for assessing stability of insert. The length of virus sequence 5' and 3' of the insert is shown. PCR products of "USER cloning" BSMV vectors are longer by 22 bp compared with restriction enzymes cloning. Not drawn to scale. **C **Stability of the BSMV-Pht1;1 and BSMV-GFP^375 ^constructs in roots of inoculated plants. cDNA prepared for qRT-PCR (panel A) was used for PCR with primers flanking the insert as shown in B. Lanes 1, 2, 3: BSMV-Pht1;1, 0 mM Pi (610 bp); 4, 5, 6: BSMV-Pht1;1, 1 mM Pi (610 bp); 7, 8, 9: BSMV-GFP^375^, 0 mM Pi (617 bp); 10, 11, 12: BSMV-GFP^375^,1 mM Pi (617 bp); 13: plasmid containing BSMVγ-PDS cassette (643 bp); 14: water control; M2: DNA marker; black arrow represents DNA fragment of 564 bp. The expected lengths for PCR products are given in brackets.

To test the potential of BSMV-mediated VIGS in barley roots, we first inserted a 368 bp fragment of the *HvPht1;1 *coding sequence into BSMV-γ-MCS, creating BSMV-γ-Pht1;1. HvPht1;1 is a plasma membrane protein mediating Pi uptake from soil and is induced in roots by P-starvation [26,27]. To induce the expression of *HvPht1;1 *we established barley plants in hydroponic culture without Pi (plants rely on Pi reserves stored in the seed) or, as a control, in culture medium containing 1 mM Pi. Barley cv. Black Hulless seeds were germinated in vermiculite soaked with the final nutrient solution for 5 days and then placed in hydroponic culture with four plants per container. One day later, the first leaf was mechanically inoculated with a mixture of RNA transcribed from clones of BSMV-α, -β and BSMV-γ-Pht1;1. As a negative control, plants were infected with BSMV carrying a 375 nt fragment of GFP (BSMV-GFP^375^). Nine days post inoculation (dpi) the entire root was harvested for analysis. Only plants showing virus symptoms at the time of harvest were included in the analysis, and all virus-infected plants growing together in one container were treated as one sample. The presence of virus in the analyzed plant roots was confirmed by RT-PCR. The expression levels of *HvPht1;1 *were determined by quantitative RT-PCR (qRT-PCR) with normalization to ubiquitin (Figure 1A). The use of 18 S RNA for normalization produced essentially the same results (data not shown). No silencing effect of BSMV-Pht1;1 was observed compared to BSMV-GFP^375^. On the contrary, *HvPht1;1 *mRNA levels were higher in plants inoculated with BSMV-Pht1;1 both in the presence and absence of Pi, but the differences are not statistically significant (*p *> 0.05). Analysis of inorganic Pi content in the harvested root samples confirmed that plants grown without Pi in the medium contained undetectable amounts of inorganic Pi (Additional file 1: Pi content in hydroponics: HvPht1;1 experiment).

Since we have found the stability of the inserted plant gene fragment to be a limiting factor for BSMV-induced gene silencing [28], we investigated the stability of the viral constructs in the barley roots using RT-PCR with primers surrounding the insert in the viral vectors (Figure 1B and 1C). The expected length for the PCR product is 610 bp from BSMV-Pht1;1 and 617 bp from BSMV-GFP^375^. As is seen in Figure 1C the PCR products were all shorter than expected, indicating that both BSMV-Pht1;1 and BSMV-GFP^375 ^are relatively unstable. The lack of silencing effect of BSMV-Pht1;1 could thus be explained by the loss of the inserted *HvPht1;1 *sequence.

**Figure 2:**
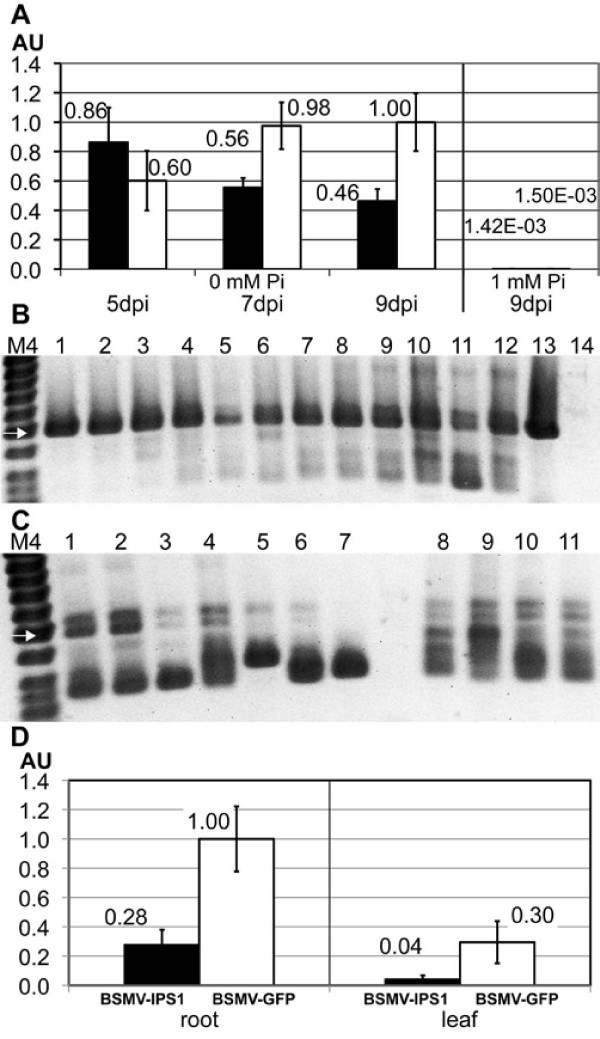
**Silencing of *HvIPS1 *in barley roots and shoots**. **A ***HvIPS1 *RNA expression levels in root tissue determined by qRT-PCR (normalization to ubiquitin). Plants inoculated with BSMV-IPS1 (black bars) or BSMV-GFP^250 ^(white bars). Plants were grown in 0 (5, 7, 9 dpi) or 1 mM Pi (9 dpi) and harvested at 5, 7, or 9 dpi. Each bar represents three samples (with one exception - only two samples for the 0 Pi, GFP, 9dpi bar). Error bars denote standard deviations. AU - Arbitrary units. **B **Stability of the BSMV-IPS1 construct in roots of inoculated plants. cDNA prepared for qRT-PCR (A) was used for PCR with primers flanking the insert. Lanes 1, 2, 3: 0 mM Pi, 5 dpi; 4, 5, 6: 0 mM Pi, 7 dpi; 7, 8, 9: 0 mM Pi, 9 dpi; 10, 11, 12: 1 mM Pi, 9 dpi; 13: plasmid containing BSMVγ-IPS1; 14: water control. M4: GeneRuler 50 bp DNA Ladder (Fermentas); white arrow represents DNA fragment of 500 bp; bands below are 400, 300, and 250 bp. **C **Stability of the BSMV-GFP^250 ^construct in roots of inoculated plants. cDNA prepared for qRT-PCR (panel A) was used for PCR with primers flanking the insert. Lanes 1, 2, 3: 0 mM Pi, 5 dpi; 4, 5, 6: 0 mM Pi, 7 dpi; 7, 8: 0 mM Pi, 9 dpi; 9, 10, 11: 1 mM Pi, 9 dpi. **D ***HvIPS1 *RNA expression levels in root and shoot tissue determined by qRT-PCR (normalization to ubiquitin). BSMV-IPS1 (black bars), BSMV-GFP^250 ^(white bars). Plant were grown in 0 mM Pi and harvested at 9 dpi. Each bar represents five samples. Error bars denote standard deviations.

In order to compare BSMV induced gene silencing in barley roots and shoots we next tried to silence a barley homologue of Arabidopsis *IPS1*. *AtIPS1 *[29] encodes a non-protein-coding RNA that is strongly induced in both roots and shoots by P-starvation. The *AtIPS1 *transcript contains a miR399 recognition site and functions as a miRNA target mimic [30]. We have identified a putative barley homologue, *HvIPS1 *(Pacak *et al*., manus in prep.). A 251 bp fragment of *HvIPS1*, downstream of the miR399 recognition site, was inserted into BSMV-γ-MCS, and seven days old plants grown under hydroponics conditions as described above were inoculated with BSMV-α, -β and either BSMV-γ-IPS1 or a BSMV-γ-GFP^250 ^control construct. At five, seven, or nine dpi, plant roots were harvested and analyzed for expression of *HvIPS1 *transcripts by qRT-PCR, again pooling all virus infected plants in a container as one sample. As is seen in Figure 2A, H*vIPS1 *expression was significantly down regulated in BSMV-IPS1 infected plants compared to BSMV-GFP^250 ^infected plants both at seven and nine dpi (t-test, *p *< 0.05). As expected, *HvIPS1 *was only expressed at detectable levels in plants grown in 0 mM Pi and not in plants grown in 1 mM Pi.

The stability of the viral constructs was again investigated by RT-PCR. PCR products of the expected length of 493 bp were the dominant products from BSMV-IPS1 infected plants at all time-points and at zero as well as high Pi content, although minor products of lower molecular weight were seen in samples from late harvested plants (Figure 2B). This suggests that the better silencing effect of BSMV-IPS1 compared to BSMV-Pht1;1 could be due to better stability of the BSMV-IPS1 construct. On the other hand, the BSMV-GFP^250 ^construct was found to be highly unstable (Figure 2C; expected length for PCR product is 492 bp), demonstrating that virus construct stability is not determined only by the length of the insert.

That BSMV-IPS1 could induce silencing of *HvIPS1 *in barley roots was confirmed in an additional experiment, this time including shoot as well as root tissue in the analysis. Leaf no. III was chosen for analysis because this leaf was previously shown to display the most extensive silencing [28]. All plants were grown in hydroponic culture with 0 mM Pi. At nine dpi plants were harvested and analyzed for *HvIPS1 *expression by qRT-PCR (Figure 2D). A significant down regulation of *HvIPS1 *was observed in both root (*p *< 0.001) and shoot (*p *< 0.05) tissue of BSMV-IPS1 infected plants compared to BSMV-GFP^250 ^infected plants. An analysis of the viral construct stability again showed that the BSMV-IPS1 construct was relatively stable over the experimental period, while BSMV-GFP^250 ^was quite unstable (data not shown).

**Figure 3:**
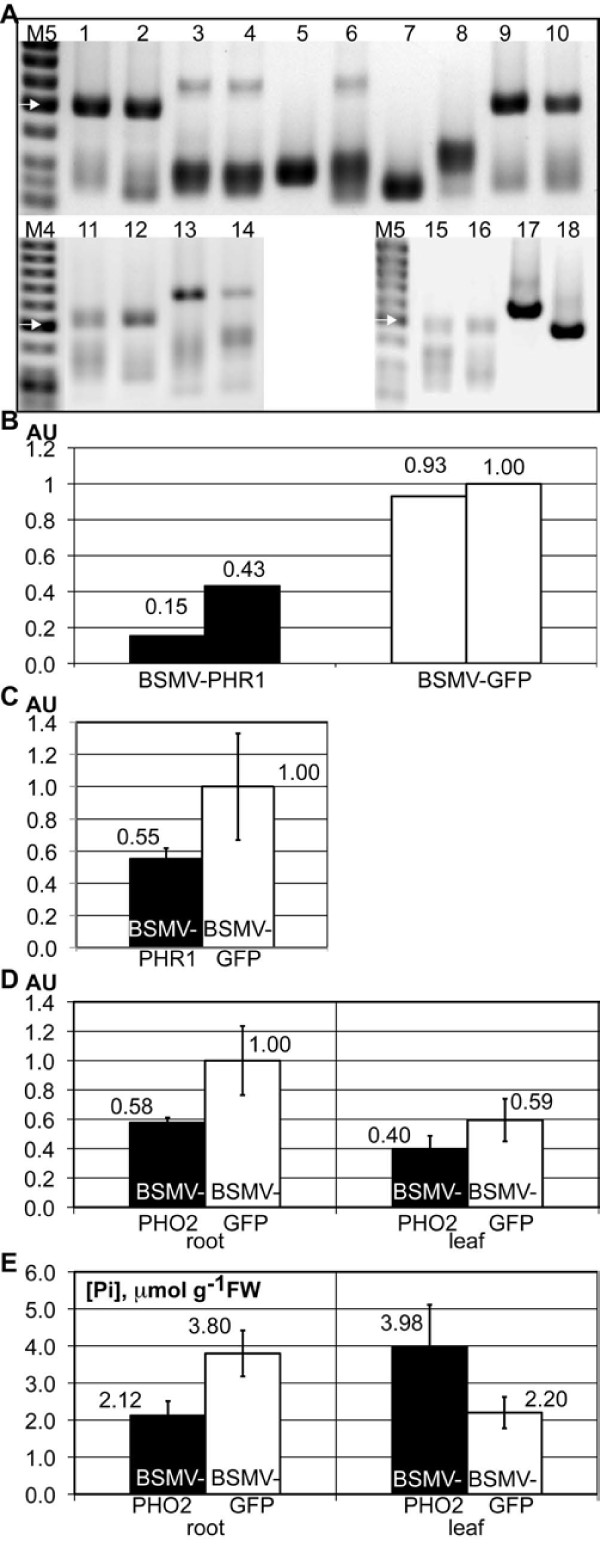
**BSMVγ constructs selected for relative stability also produce successful gene silencing in barley roots**. **A **RT-PCR analysis of the stability of BSMVγ constructs in barley roots. Expected lengths for the PCR products are presented in brackets. Lanes 1, 2: BSMV-IPS1 (493 bp); 3, 4: BSMV-Pht1;1 (610 bp); 5, 6: BSMV-Pht1;4 (616 bp); 7, 8: BSMV-Pht1;7 (623 bp); 9, 10: BSMV-PHR1 (495 bp); 11, 12: BSMV-PHO2^247 ^(511 bp); 13, 14: BSMV-PHO2^387 ^(651 bp); 15, 16: GFP^250 ^(492 bp); 17: control plasmid carrying BSMVγ-GFP^375 ^(617 bp); 18: control plasmid carrying BSMVγ-IPS1 (493 bp); M4: GeneRuler 50 bp DNA Ladder (Fermentas); M5: O'GeneRuler 50bp DNA Ladder (Fermentas). White arrow represents DNA fragment of 500 bp; bands below are 400, 300, and 250 bp. **B and C ***HvPHR1 *expression levels in root tissue determined by qRT-PCR, normalization to 18 S rRNA. BSMV-PHR1 (black bars), BSMV-GFP^250 ^(white bars). Samples in B are identical to those shown in A, lanes 9, 10 and 15,16. In C, each bar represents five independent samples. AU - arbitrary units; error bars denote standard deviations. **D ***HvPHO2 *expression levels in root and leaf tissue determined by qRT-PCR, normalization to 18 S rRNA. BSMV-PHO2^247 ^(black bars), BSMV-GFP^250 ^(white bars). **E **As D, but bars represent Pi content in inoculated plants (in μmol Pi/g of fresh weight).

The successful silencing obtained with BSMV-IPS1 demonstrates that BSMV can be used to induce gene silencing in barley roots. However, instability of the viral vector may be a more severe problem for silencing of genes in roots compared to leaves, since it is more difficult to limit the analysis to a "window of optimal silencing" in roots than in leaves, where the third or fourth leaf is often chosen for analysis. Therefore, screening virus constructs for stability may be advisable. We inoculated five new BSMV constructs carrying fragments of different root expressed barley genes to barley cv. Black Hulless plants grown in garden soil under Pi-replete conditions: Pi transporter no 4 (*HvPht1;4*, 374 bp fragment), Pi transporter no 7 (*HvPht1;7*, 381 bp fragment), and putative homologues of the *PHR1 *and *PHO2 *genes from Arabidopsis (*HvPHR1*, 253 bp, and *HvPHO2*, 247 bp and 387 bp fragments). The previously used BSMV-Pht1;1, BSMV-IPS1, and BSMV-GFP^250 ^constructs were also included in order to test whether the artificial hydroponics conditions had influenced the stability. Two pots were used for each construct; each pot contained three plants, where all virus-infected plants were treated as one sample. At nine dpi, the roots were harvested and the stability of the BSMV-γ constructs was analysed by RT-PCR (Figure 3A). As in the hydroponics experiments, the BSMV-IPS1 construct was found to be relatively stable and the BSMV-Pht1;1 and BSMV-GFP^250 ^constructs highly unstable. Furthermore, BSMV-PHR1, BSMV-PHO2^247 ^and BSMV-PHO2^387 ^appeared relatively stable, while BSMV-Pht1;4 and BSMV-Pht1;7 appeared as unstable as BSMV-Pht1;1.

The apparent stability of the BSMV-PHR1 and BSMV-PHO2 constructs prompted us to test the silencing capacity of these constructs in barley roots. First, *HvPHR1 *expression in the two BSMV-PHR1 infected root samples used in Figure 3A (lanes 9 and 10) was analyzed by qRT-PCR and compared to the expression in the two BSMV-GFP^250 ^infected samples (lanes 15 and 16). The result suggested that the BSMV-PHR1 construct was indeed able to induce efficient silencing (Figure 3B). To confirm this, the experiment was repeated with five independent samples (each consisting of three plants in a pot with garden soil) for both BSMV-PHR1 and BSMV-GFP^250^. Seven days old barley plants were inoculated and after nine days the roots were harvested. Again, expression of *HvPHR1 *was significantly down regulated (*p *< 0.05) by infection with BSMV-PHR1 (Figure 3C). A stability analysis confirmed that the BSMV-PHR1 construct was highly stable (data not shown). Next, BSMV-PHO2^247 ^and BSMV-GFP^250 ^were both inoculated to seven pots containing three 7-days old barley plants each. At 9 dpi roots as well as the third leaf were harvested and *HvPHO2 *expression was analysed by qRT-PCR. A significant reduction in *HvPHO2 *mRNA levels was seen both in leaves (*p *< 0.02) and in roots (*p *< 0.001) infected with BSMV-PHO2^247 ^relative to BSMV-GFP^250 ^(Figure 3D). Since the homologous gene in Arabidopsis is a negative regulator of Pi translocation from roots to shoots [31-33], we measured the free Pi content in the root and leaf samples (Figure 3E). Pi content was significantly up-regulated in shoots (*p *< 0.003) and downregulated in roots (*p *< 0.001) in BSMV-PHO2^247 ^infected plants relative to BSMV-GFP^250 ^infected plants. This suggests that *HvPHO2 *may play a similar role in barley as *AtPHO2 *in Arabidopsis.

### Short inverted repeats are not generally superior to sense fragments

**Figure 4:**
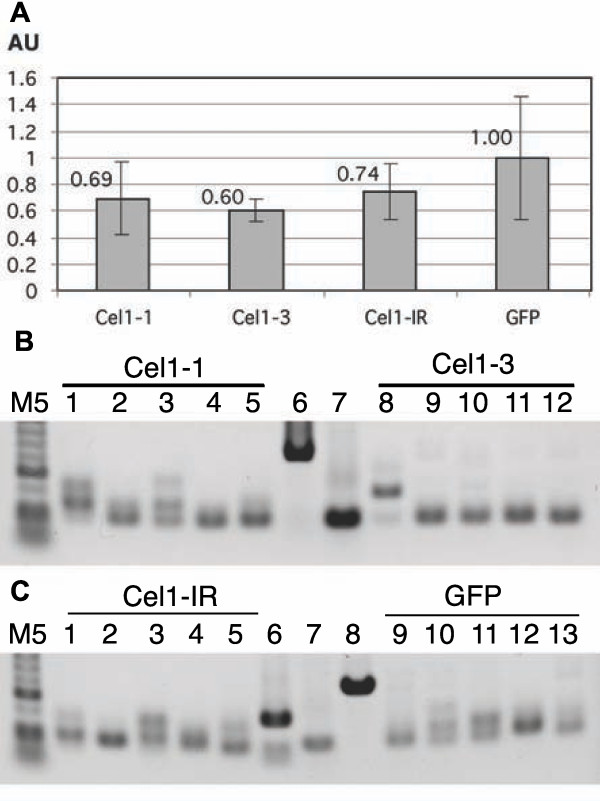
**Short inverted repeats are not inherently more stable or efficient for BSMV induced VIGS in barley roots**. **A ***HvCel1 *RNA expression levels in root tissue determined by qRT-PCR (normalization to 18 S rRNA). Plants were inoculated with either BSMV-Cel1-1, BSMV-Cel1-3, BSMV-Cel1-IR, or BSMV-GFP^375^. Each bar represents five samples, each consisting of one plant in a pot. Error bars denote standard deviations. AU - Arbitrary units. **B **and **C **Stability of the BSMV constructs in roots of infected plants. cDNA prepared for qRT-PCR (panel A) was used for PCR with primers flanking the insert. B lanes 1-5: plants infected with BSMV-Cel1-1 (643 bp); lane 6: plasmid containing BSMV-Cel1-1 (643 bp); lane 7: plasmid containing BSMV without insert (242 bp); lanes 8-12: plants infected with BSMV-Cel1-3 (641 bp). C lanes 1-5: plants infected with BSMV-Cel1-IR (388 bp); lane 6: plasmid containing BSMV-Cel1-IR (388 bp); lane 7: plasmid containing BSMV without insert (242 bp); lane 8: plasmid containing BSMV-GFP^375 ^(617 bp); lanes 9-12: plants infected with BSMV-GFP^375 ^(617 bp). M5: O'Gene Ruler 50 bp DNA ladder (Fermentas). Expected lengths for the PCR products are given in brackets.

Short inverted repeats have been reported to produce more efficient silencing when inserted into a BSMV vector as compared to longer sense or antisense fragments [34]. Since shorter inserts tend to be more stably retained in the BSMV vector than longer [28] we reasoned that short inverted repeats might be generally more stably retained and therefore more efficient at inducing silencing. To test this assumption, we compared the silencing efficiency of two non-overlapping, 401 bp fragments (Cel1-1 and Cel1-3) of the barley *HvCel1 *gene (AB040769), with a 58bp inverted repeat derived from within the Cel1-3 region (termed Cel1-IR). *HvCel1 *is a membrane-anchored endo-β-1,4-glucanase and is expressed both in leaves and roots [35], and the homologous *Kor1 *gene has been successfully silenced in pea roots by VIGS using a pea early browning virus vector [11]. The first leaf of barley cv. Black Hulless plants grown in soil was inoculated with BSMV-α, -β and either BSMV-γ-Cel1-1, BSMV-γ-Cel1-3, BSMV-γ-Cel1-IR, or the BSMV-γ-GFP^375 ^control construct. At nine dpi, plant roots were harvested and analyzed for expression of *HvCel1 *transcripts by qRT-PCR. As is apparent from Figure 4a, n one of the constructs induced significant silencing (*p *> 0.05). In agreement with this, a stability analysis by RT-PCR showed that all the BSMVγ-Cel1 constructs were highly unstable (Figure 4b and c). Thus, the construction of short inverted repeats does not necessarily result in more stable or more efficient silencing inducing BSMV constructs.

### VIGS in *B. distachyon*

To test the potential of BSMV for VIGS in *B. distachyon *we tried to silence the phytoene desaturase gene (*PDS*). *PDS *is often used as a visual reporter for VIGS since its silencing produces easily visible chlorophyll photobleaching [36]. A 433 nt fragment of *PDS *from *B. distachyon *was first produced by RT-PCR using primers designed based on alignment of *PDS *sequences from other monocot species. The sequence of the cloned product (filed as Genbank acc. No. HM755884) showed 92% nt sequence identity to the corresponding fragments of barley, wheat and rice *PDS *(Additional file 2: Alignment of *PDS *sequences). A 300 nt internal fragment of *BdPDS *was then amplified by PCR and inserted into BSMV. For these experiments, two modified vector versions called BSMVγ-USER and BSMVγ-T4 allowing ligation-free cloning were employed (see below for description of the vectors).

**Figure 5:**
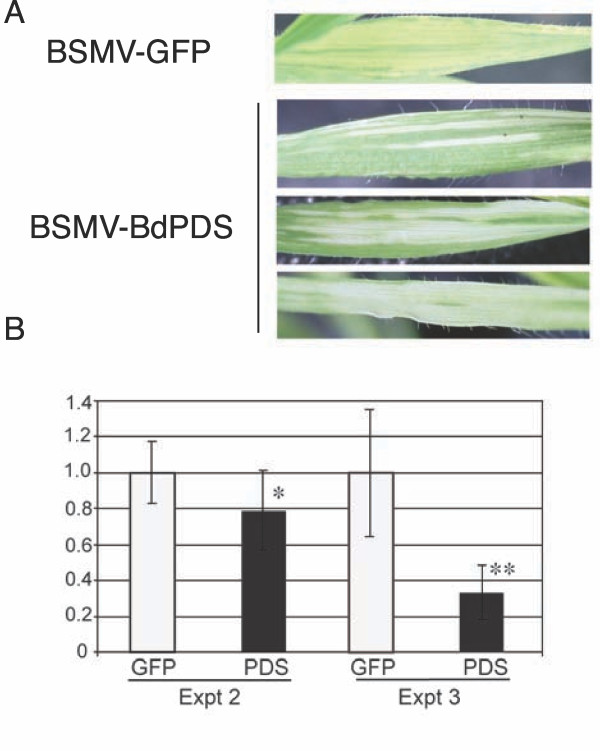
**Silencing of *BdPDS *in *B. distachyon *leaves A **Leaves of *B. distachyon *infected with BSMV-GFP^375 ^or BSMV-BdPDS. **B ***BdPDS *RNA expression levels in experiments 2 and 3 (Table 1) determined by qRT-PCR (normalization to 18 S rRNA). Plants were inoculated with either BSMV-BdPDS (black bars) or BSMV-GFP^375 ^(white bars). From left to right, the bars represent 16, 13, 6 and 6 samples, respectively. Error bars denote standard deviations. AU - Arbitrary units. Significantly differences: * (*p *< 0.01), ** (*p *< 0.0001).

Two weeks after sowing, leaves of *B. distachyon *line Bd21-3 plants were inoculated with BSMV-α, -β, and either BSMV-γ-BdPDS or BSMV-γ-GFP^375^. The appearence of virus symptoms and/or *PDS *silencing in three independent experiments is shown in Table 1. *PDS *silencing was scored visually as development of photobleaching. On average more than half of the inoculated plants developed symptoms of silencing, or two-thirds of those that became infected. The intensity of photobleaching varied between plants, from small white stripes to almost complete bleaching of one or more leaves (Figure 5A). Plants infected with BSMV-GFP^375 ^never developed photobleaching but only the yellow stripes also typical of BSMV infection in barley and wheat. In order to quantify the silencing effect, all the systemically infected leaves from all plants showing virus symptoms were harvested at 17 dpi in experiment 2. Analysis by quantitative qRT-PCR showed a modest but statistically significant reduction in *PDS *mRNA levels in plants infected with BSMV-BdPDS compared to BSMV-GFP^375 ^(*p *< 0.01) (Figure 5B). The analysis was repeated in experiment 3, where systemically infected leaves from six plants of each group were harvested at 13 dpi. In this experiment, leaves showing photobleaching were preferentially chosen for harvest. qRT-PCR again showed a reduction in PDS mRNA levels in plants infected with BSMV-BdPDS compared to BSMV-GFP^375^, the difference being highly significant (*p *< 0.0001) (Figure 5B). These results suggest that BSMV can be a useful tool for gene analysis in *B. distachyon*.

**Table 1:** PDS silencing in B. distachyon

	Symptoms	Plants with symptoms
*Expt. 1a*	S	5/12
**PDS**	V	6/12

*Expt. 1b*	S	4/10
**PDS**	V	5/10

*Expt. 2*	S	10/22
**PDS**	V	3/22

*Expt. 2*	S	0/22
**GFP**	V	16/22

*Expt. 3*	S	20/28
**PDS**	V	5/28

*Expt. 3*	S	0/28
**GFP**	V	23/28

Virus symptoms and PDS silencing in *B. distachyon *leaves 3 weeks after inoculation with BSMV-BdPDS or BSMV-GFP^375^.

S: plants displaying photobleaching (silencing) as well as virus symptoms. V: plants displaying virus symptoms only. Plants in experiment 1b were inoculated with BSMV-T4-BdPDS, otherwise BSMV-USER-BdPDS was used. Plants were inoculated at 11 (expt. 1), 14 (expt. 2), or 19 (expt. 3) days after sowing.

To test for infectivity and thus silencing potential of BSMV in *B. distachyon *roots, *B. distachyon *plants inoculated with the empty BSMV vector (BSMV-MCS) were harvested at 18 dpi and the presence of BMSV coat protein was determined separately in leaves and roots by double-antibody sandwich enzyme-linked immunosorbent assay (DAS-ELISA) (Additional file 3: BSMV in *B. distachyon *leaves and roots). In all 12 plants tested, strong positive reactions were obtained from both leaf and root samples, indicating that BSMV has potential as a VIGS vector in roots as well as leaves of this species.

### VIGS in oat

VIGS experiments have not previously been reported for oat. The existing BSMV-based vectors are all based on the ND18 strain, which does not infect oat [37]. However, infectivity on oat has been reported for the CV42 strain of BSMV, as well as for pseudorecombinants of the two strains containing the CV42 α component [37]. We therefore decided to test the silencing potential of the BSMV VIGS vector in oat when combined with the BSMV^CV42 ^α component. In this paper, "BSMV" and "BSMV^ND18^" both refer to ND18-derived virus clones while "BSMV^CV42^" refers to clones derived from strain CV42 [38]. In a first experiment, the susceptibility of a range of oat cultivars to different BSMV component combinations was investigated using visual observation of virus symptoms as well as detection of the virus coat protein by DAS-ELISA.

**Figure 6:**
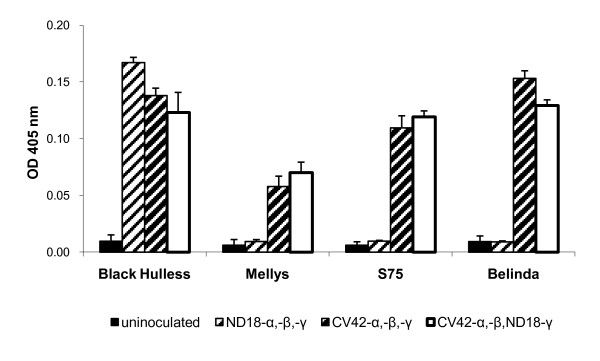
**Detection of BSMV coat protein by ELISA**. Barley (Black Hulless) and oat (Mellys, S75, Belinda) plants were left uninoculated or inoculated with BSMV^CV42^-α, -β and -γ, BSMV^ND18^-α, -β and -γ or a combination of BSMV^CV42^-α, -β and BSMV^ND18^-γ. Extracts of the third leaf, harvested at 14 dpi, were assayed by DAS-ELISA to detect BSMV coat protein. The average OD_405 _values of five plants from each group are shown. Error bars indicate standard deviations.

The first leaf of plants of the diploid oat species *A. strigosa *cultivars S75 and Melly, the hexaploid *A. sativa *cv. Belinda and barley cv. Black Hulless was mechanically inoculated with RNA transcribed from clones of BSMV^CV42^-α, -β and -γ; BSMV^ND18^-α, -β and -γ, or a combination of BSMV^CV42^-α, -β, and BSMV^ND18^-γ. All combinations produced systemic mosaic virus symptoms in barley plants at five to seven dpi. Leaves of oat plants inoculated only with BSMV components showed no virus symptoms, but virus symptoms were observable in oat plants inoculated with BSMV^CV42 ^components alone or with BSMV^CV42^-α, -β, and BSMV^ND18^-γ at seven to 10 dpi. All inoculated S75 and Belinda plants showed similar virus symptoms, while Melly plants showed weaker symptoms and only on some plants. By 14 dpi the third leaf was harvested and the presence of BSMV coat protein was detected by DAS-ELISA (Figure 6). Barley plants tested positive with all three BSMV component combinations. In oat plants, similar levels of coat protein were detected after inoculation with either BSMV^CV42^-α, -β, -γ or BSMV^CV42^-α, -β, BSMV^ND18^-γ, but none was detected after inoculation with BSMV^ND18^-α, -β, -γ. Less coat protein was detected in Melly plants compared to S75 and Belinda, consistent with the visual observations. The experiment was repeated with the inclusion of more oat cultivars (Table 2). In most cultivars, the pseudorecombinant BSMV^CV42^-α, -β, BSMV^ND18^-γ produced similar levels of coat protein as the pure CV42 strain. As expected, no oat cultivar was susceptible to the pure ND18 BSMV strain. The diploid *A. strigosa *cv. S75 and the hexaploid *A. sativa *cv. Belinda were chosen for further silencing experiments.

**Table 2:** BSMV accumulation in oat cultivars

	BSMV coat protein detection		
	
Cultivars	ND18-α-β-γ	CV42-α-β,-γ	CV42-α-β, ND18-γ
Mellys	**-**	++	++
S75	**-**	+++	+++
Belinda	**-**	+++	+++
Triple Crown	**-**	++	+
Sang	**-**	+++	+
Mathilda	**-**	+++	++
Kerstin	**-**	+++	+++
Ingeborg	**-**	+++	+++
Cilla	**-**	+++	+++
Betania	**-**	+++	+++
Avery	**-**	++	+++
A sterilis 11089		++	+++
A strigosa 14522		++	+++
A strigosa 8768		+++	+++
A strigosa 8770		++	++
A strigosa 5399		+++	++
Black Hulless	+++	+++	+++

Different cultivars of oat and barley were inoculated with BSMV^CV42^-α, -β and -γ, BSMV^ND18^-α, -β and -γ, or a combination of BSMV^CV42^-α, -β and BSMV^ND18^-γ. BSMV coat protein was detected by DAS-ELISA in extracts from the third leaf at 14 dpi. Coat protein levels were grouped as follows: - OD405 around 0.01, similar to healthy plants; + 0.03 < OD405 < 0.05; ++ 0.05 < OD405 < 0.10; +++ 0.10 < OD405.

The *PDS *gene was chosen as a first target of silencing, but the oat *PDS *gene (*AsPDS*) has not been reported previously. To obtain fragments of *AsPDS *we performed RT-PCR on *A. strigosa *cv. S75 mRNA using primers designed from conserved nucleotide sequences of monocot PDS mRNAs. Two partly overlapping fragments, *AsPDS1 *(422bp) and *AsPDS2 *(401bp) were obtained and cloned into the ND18-based BSMV-MCS vector. The combined sequence of the fragments (filed as GenBank acc. No. HM755676) showed 92 and 93% nt identity to barley and wheat *PDS *genes, respectively, with several regions of 100% identity over more than 25 nt (Additional file 2: Alignment of *PDS *sequences).

**Figure 7:**
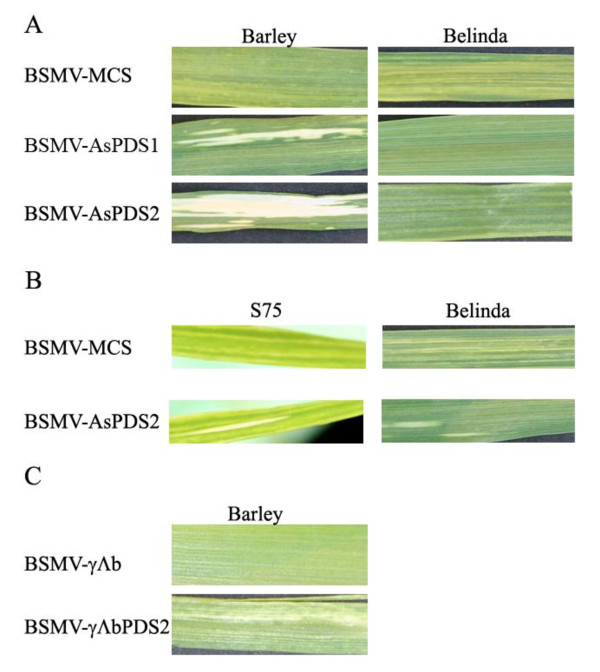
**Virus symptoms and silencing phenotype in oat compared to barley**. Barley cv. Black Hulless, oat (*A. sativa*) cv. Belinda and diploid oat (*A. strigosa*) cv. S75 were inoculated with BSMV^CV42^-α, -β, and different BSMV^ND18^-γ components as indicated. Photos were taken at 14 dpi.

To test the silencing effect of the BSMV-AsPDS vectors, the first leaf of ten-days-old *A. strigosa *cv. S75 and seven-days-old *A. sativa *cv. Belinda and barley cv. Black Hulless plants was mechanically inoculated with RNA transcribed from clones of BSMV^CV42 ^-α and -β together with BSMV^ND18^-γ-AsPDS1, BSMV^ND18^-γ-AsPDS2 or the empty vector BSMV^ND18^-γ-MCS. Plants inoculated with BSMV-MCS showed yellow mosaic virus symptoms from seven (barley) or 10 (oat) dpi (shown in Figure 7 at 14 dpi). The expected white photobleaching pattern in leaves was observable on barley leaves after 10 dpi with either BSMV-AsPDS1 or BSMV-AsPDS2. In plants inoculated with BSMV-AsPDS2 a higher percentage of the leaf area was photobleached, suggesting a stronger silencing effect. In the first experiments no photobleaching was observed in oat cultivars inoculated with BSMV-AsPDS1 or BSMV-AsPDS2. In addition no typical virus symptoms were visible. Since the infection frequencies with oat tended to be lower than with barley, in further experiments the inoculation method was optimized. This included performing RNA inoculation with a cotton swap instead of a gloved finger, and covering the plants with plastic for a week after inoculation to increase the humidity. Oat plants inoculated under optimized conditions recovered much better from the inoculation procedure than before, but infection frequencies were still lower (~50%) than with barley (~100%). Barley plants showed the same phenotype as described before and seen in Figure 7A. In S75 and Belinda plants inoculated with BSMV-MCS, virus symptoms appeared by 10 to 14 dpi (Figure 7B). Oat plants inoculated with BSMV-AsPDS1 again showed no silencing phenotype and only a few plants showed virus symptoms, which were weaker than on barley (not shown). In a few S75 and Belinda plants inoculated with BSMV-AsPDS2, white stripes indicative of *PDS *silencing were observed (Figure 7B). Compared to inoculated barley leaves the silencing phenotype was much weaker. Tests using DAS-ELISA confirmed that oat plants without virus symptoms also had no detectable BSMV coat protein (data not shown).

To improve silencing, we tested another variant of BSMV-γ, which was used previously in silencing experiments in wheat [39]. In this ND18-based BSMV-γ variant the start codon of the γb open-reading frame was modified to create a *Bam*HI site and γb expression was blocked (designated in the following as BSMV-γΔb). By insertion of the AsPDS2 fragment into the γΔb-vector, a part of the γb protein was deleted and a vector γΔbAsPDS2 was obtained. Belinda and S75 plants showed neither virus symptoms nor silencing when inoculated with BSMV^CV42 ^α and β together with BSMV-γΔbAsPDS2 or BSMV-γΔb. In barley plants inoculated with these virus vectors both photobleached areas and virus symptoms were observed (Figure 7C; BSMV-γΔbPDS2 and BSMV-γΔb). Compared to BSMV-AsPDS2 and BSMV-MCS visual determination revealed a reduced effect for the γΔb vector variants.

Increased silencing efficiency in barley has previously been reported by using a BSMV-β component with a deleted coat protein (βΔβa [3]). In oat plants no phenotypical difference was observed when using the BSMV-βΔβa component instead of BSMV^CV42^-β (data not shown). Also the use of BSMV^ND18^-β instead of BSMV^CV42^-β had no influence on virus infectivity or silencing effects (data not shown). The use of BSMV-βΔβa in barley plants was associated by extensive necrosis as reported before [28].

To ensure that the poor gene silencing effect in oat was not specific for the *PDS *gene, another round of experiments was conducted targeting a presumed oat homologue of the *HvCel1 *gene. Using primers designed from an oat EST sequence (Genbank acc. No. CN821108) two non-overlapping 399 bp fragments, AsCel1-2 and AsCel1-3, were produced by RT-PCR from *A. strigosa *cv. S75 mRNA and cloned into the ND18-based BSMV-MCS vector. The combined sequence of the fragments (filed as GenBank acc. No. HM755677) showed 89% nt identity to *HvCel1 *(data not shown). *A. strigosa *cv. S75 plants were inoculated as described above with BSMV^CV42 ^-α and -β together with BSMV^ND18^-γ-AsCel1-2, BSMV^ND18^-γ-AsCel1-3 or the control vector BSMV^ND18^-γ-GFP^375^. At 12 dpi, leaves displaying virus symptoms were harvested and expression of *AsCel1 *was determined by qRT-PCR (see Additional file 4: Lack of silencing of *AsCel1 *in oat shoots). No significant differences between the three groups of plants were obtained.

In summary, only very weak signs of silencing were obtained in two different oat species and with two different target genes, indicating that BSMV is not a promising gene silencing vector for oat.

### BSMV vectors adapted for ligation-free cloning

**Figure 8:**
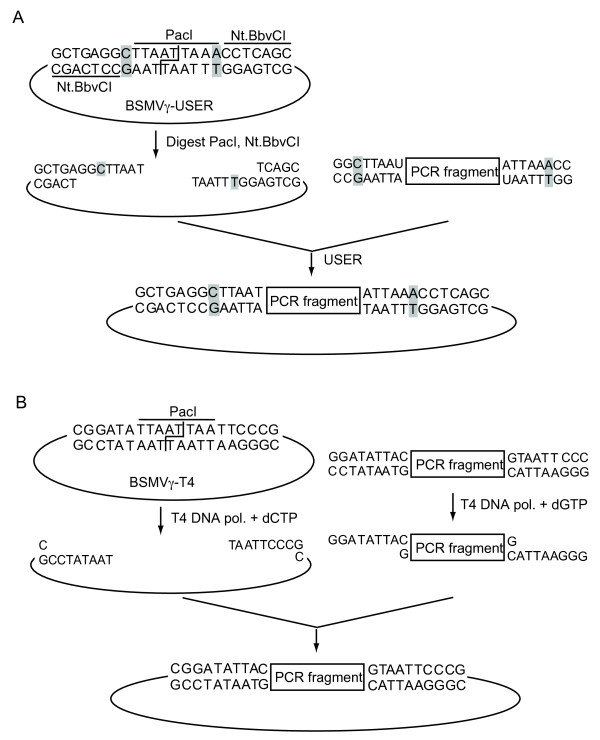
**BSMV vectors adapted for ligation-free cloning**. **A **Cloning of inserts into BSMVγ-USER. See text for details. **B **Cloning of inserts into BSMVγ-T4. See text for details.

The two BSMV vectors used in most studies to date contain either *Pac*I and *Not*I sites [3] or *Sma*I, *Pac*I and *Bam*HI sites [28] for introduction of gene fragments into the gamma genome component. To speed up the production of new VIGS constructs we have constructed two modified vectors enabling ligation-free cloning. The first vector, BSMV-USER, is based on the USER cloning strategy as described by Nour-Eldin et al. [40]. The *Pac*I and *Bam*HI sites of the original vector, BSMV-MCS [28] were replaced by a single *Pac*I site surrounded by Nt.BbvCI recognition sites. The vector is prepared for insertion of gene fragments by cleavage with *Pac*I followed by treatment with the nicking enzyme Nt.BbvCI. This creates 8 nt 3' overhangs and directional cloning is enabled by the presence of a single cytosine residue upstream of the *Pac*I site and a single adenosine residue downstream of the *Pac*I site (Figure 8A). After digestion the vector can be stored in aliquots at -20°C for at least a year. Fragments to be inserted are produced by PCR with primers whose 5' ends contain 7 nts corresponding to the vector overhangs followed by a uracil residue. The unpurified PCR product is mixed with an aliquot of the stored vector and 1 U of the USER™enzyme that excises the uracil residue, leaving a gap. After 40 min incubation the mixture is used directly for transformation of bacterial cells. The second vector version, BSMV-T4, is based on the use of T4 DNA polymerase for generating long 5' overhangs, similar to the strategy described for the tobacco rattle virus vector TRV-LIC [41]. The *Pac*I/*Bam*HI sites of the original vector were replaced by a single *Pac*I site surrounded by five non-C nucleotides followed by a C (Figure 8B). The vector is prepared for insertion of gene fragments by cleavage with *Pac*I followed by treatment with T4 DNA polymerase in the presence of dCTP, creating 8 nt 5' overhangs. After heat-inactivation, this vector can also be stored at -20°C for later use. Fragments to be inserted are produced by PCR with primers whose 5' ends contain 8 nts complementary to the vector overhangs. The PCR product is column purified, treated with T4 DNA polymerase in the presence of only dGTP, heat-inactivated, incubated briefly with an aliquot of prepared vector and used directly for transformation of bacterial cells.

The cloning efficiencies for the two vectors were tested by insertion of the 300 nt *BdPDS *fragment (see above) amplified by PCR using primers designed for either the BSMV-USER or the BSMV-T4 vector. After transformation, more than a hundred colonies were produced from both vectors, and 12 colonies of each were randomly selected. All the BSMVγ-USER-BdPDS clones and six of the BSMVγ-T4-BdPDS clones were found to contain the correct insert. The BSMVγ-USER-BdPDS construct was used for most of the *B. distachyon *VIGS experiments reported above; one experiment employed the BSMVγ-T4-BdPDS construct. No difference in efficiency of the two constructs was observed. The BSMVγ-USER vector has subsequently been used for preparation of several different VIGS constructs in our laboratory, including the BSMV-PHO2^247^, BSMV-PHO2^387 ^and BSMV-γ-Cel1-IR constructs used for silencing experiments in barley roots (see above). In all cases, large numbers of colonies were obtained, all of which contained the desired insert. The inverted repeat of BSMV-Cel1-IR was made by simultaneous insertion [40] of the sense and the antisense fragments as PCR products into the BSMV-USER vector. Again 15 out of 15 colonies tested contained the expected insert, demonstrating the high efficiency of the USER cloning method.

## Discussion

We here present the first evidence of virus induced gene silencing in roots of a monocotyledonous plant species. The transcript levels of *HvIPS1, HvPHR1 *or *HvPHO2 *were significantly decreased in the roots of barley plants infected with BSMV-IPS1, BSMV-PHR1 or BSMV-PHO2^247 ^relative to plants infected with the control construct BSMV-GFP^250^. On the other hand, our attempts at silencing the *HvPht1;1 *and *HvCel1 *genes failed. The difference in silencing efficiency for the five genes could be related to differences in experimental conditions or to characteristics of the silencing inducing sequences, the viral constructs, the target genes, or a combination hereof.

Growth conditions, and in particular temperature, are known to be important for the success of VIGS experiments [42]. However all experiments reported here were performed in growth chambers at the same temperature, 20°C. Successful gene silencing in roots was observed under hydroponic growth conditions (*HvIPS1*) as well as in plants grown in soil (*HvPHR1 *and *HvPHO2*), and unsuccessful VIGS results were also obtained under both conditions (*HvPht1;1 *and *HvCel1*, respectively). Furthermore, the successful silencing of *HvIPS1 *was obtained under P-starvation conditions, while the silencing of *HvPHR1 *and *HvPHO2 *was achieved under Pi repleteness conditions. Thus the different outcomes with these five target genes seem unrelated to the growth conditions.

Previous studies have indicated that the plant Dicer-like proteins producing siRNAs from dsRNA show a preference for GC rich regions [43,44], suggesting that GC rich gene fragments would be more likely to induce successful VIGS. An analysis of the silencing inducing fragments used here failed to show any correlation between GC content and silencing, with the successful *HvIPS1 *fragment having the lowest GC content (41.8%) and the unsuccessful *HvPht1;1 *fragment the highest (58.1%). See also Additional file 5: GC content in silencing fragments.

The length of the silencing inducing sequence might also influence silencing efficiency. Fragments as short as 33 nt inserted into potato virus X were shown to induce silencing of the *PDS *gene in *Nicotiana benthamiana*, but a 368 nt fragment produced stronger silencing [45]. In previous VIGS experiments with BSMV in barley, efficient silencing of the *PDS *gene has been obtained with fragments ranging from 120 nt to 1215 nt [3,4,28]. In the experiments reported here, the successful silencing inducing sequences were 247, 251 or 253 nt in length, respectively, while the unsuccessful sequences were 368 or 401 nt in length or an inverted repeat of 58 nt. Thus, the length of the silencing inducing sequence does not seem directly correlated to the efficiency of silencing.

The length of the sequence inserted into a viral vector may however indirectly impact on the silencing efficiency by influencing the stability of the modified virus. Many viral vectors show a tendency to loose the inserted sequence over time [8,46,47]. We have previously shown that the transient nature of BSMV-induced gene silencing is related to instability of the modified virus; the shortest insert tested (128 nt) was most stable, with instability increasing progressively with increasing length up to the longest tested (584 nt) [28]. This prompted us to investigate the stability of the viral constructs used here. The successful construct BSMV-IPS1 proved to be relatively stable, while the unsuccessful BSMV-Pht1;1 was highly unstable. A test of five more constructs, BSMV-Pht1;4, BSMV-Pht1;7, BSMV-PHR1, BSMV-PHO2^247 ^and BSMV-PHO2^387^, indicated that BSMV-PHR1 and the two BSMV-PHO2 constructs had a stability similar to BSMV-IPS1, and indeed both BSMV-PHR1 and BSMV-PHO2^247 ^were able to induce significant silencing of their target genes in barley roots. On the other hand, three further viral constructs only inducing weak silencing of their target, *HvCel1*, were all highly unstable. Thus, the stability of the individual viral constructs can account for the differences in silencing efficiency observed.

The reason for the great variation in stability between individual viral constructs is unclear. The length of the insert may be of importance, since three of the most stable constructs, BSMV-IPS1, BSMV-HvPHR1 and BSMV-PHO2^247^, also carried the shortest of the simple sense inserts, and an inverse correlation between stability and insert length has previously been shown for the *PDS *gene [28]. However, the BSMV-GFP^250 ^construct was highly unstable, while BSMV-PHO2^387 ^appeared relatively stable, demonstrating that length is not the sole determinant of stability. Possibly, secondary structure of the insert may interfere with steps in the viral infection cycle such as replication or assembly of viral particles. In agreement with this hypothesis, the inverted repeat construct, BSMV-Cel1-IR, was highly unstable despite carrying an insert of only 124 nt in total length. The expected hairpin-loop structure of the inverted repeat may be especially prone to interfere with viral infection steps. The secondary structures of the other inserts in the viral genome context are more difficult to predict, and other features such as local GC content or cryptic motifs might also interfere with the viral infection cycle. A negative effect of the insert on virus fitness would favour the emergence of viral constructs with deletions of the insert. In a previous VIGS study in pea, different silencing efficiencies were obtained when two different *Nin *gene fragments of equal size were inserted into a *Pea early browning virus*-based vector. The difference was attributed to differences in viral fitness, since the viral construct inducing the most efficient silencing also accumulated in larger amounts in infected plants [12].

The efficiency of silencing can also be influenced by characteristics of the target genes, such as accessibility of target sequences for siRNAs, possibly related to target RNA secondary structure [48,49], or feed-back regulation leading to increased transcription when mRNA levels are reduced by RNA silencing [50,51]. Genes with high transcription rates and short mRNA half-lives will also appear more refractory to RNAi than genes with slower natural turn-over [52]. We cannot rule out that such factors may also contribute to the different silencing efficiencies we observe.

The *HvIPS1 *and *HvPHO2 *genes are expressed in both leaf and root tissues, and BSMV-IPS1 and BSMV-PHO2^247 ^induced similar levels of silencing in both organs. The leaf samples analyzed were taken from leaf no. III where silencing is expected to be most strongly manifested [28]. The root samples were taken after crushing the entire root and thus represent an average of all parts of the root. The significant silencing seen in root tissue with the more stable BSMV constructs suggests that BSMV can induce gene silencing in roots at least as strongly as in leaves. However, the sampling difficulties in roots put greater demands on the silencing efficiency/stability of the virus.

We measured the free Pi contents in the root samples of plants grown under hydroponic conditions. This confirmed that the 0 mM Pi growth conditions indeed led to extremely low levels of free Pi in the plant roots (Additional file 1: Pi content in hydroponics: *HvPht1;1 *experiment). Silencing of the *HvIPS1 *gene might have been expected to lead to increased Pi levels, if this gene plays a similar role in Pi regulation in barley as previously shown for the homologous gene in Arabidopsis [30]. However the differences observed were not statistically significant due to the extremely low Pi content of plants grown without addition of Pi (Additional file 6: Pi content in hydroponics: *HvIPS1 *experiment). Further studies including more sensitive (e.g. P-33 autoradiography) Pi measurements and/or more sophisticated Pi addition regimes will be necessary to establish the impact of *HvIPS1 *silencing on the Pi uptake in barley. On the other hand, silencing of *HvPHO2 *under Pi repleteness conditions led to a significant increase in leaf Pi and a similar decrease in root Pi content (Figure 3E). This is in agreement with previous studies in Arabidopsis, where *pho2 *mutants show increased root-to-shoot translocation of Pi and excessive accumulation of Pi in leaf tissues [31-33]. Our data indicate that the barley *PHO2 *homologue has similar roles in Pi regulation in barley as previously shown for *PHO2 *genes in Arabidopsis, and that BSMV-mediated VIGS can be used to explore the roles of barley genes in nutrient acquisition and distribution.

Our VIGS experiments in *B. distachyon *were successful, in that two out of three plants infected with BSMV-BdPDS developed photobleaching accompagnied by significant reductions in *BdPDS *mRNA levels (Table 1 Figure 5). These data suggest that BSMV could be a very useful tool for functional genomics in this new model species. We have not attempted gene silencing in *B. distachyon *roots, but since BSMV was found to infect the roots efficiently (Additional file 3) it is likely that this vector will also be useful for VIGS in *B. distachyon *roots. The experiments reported here were conducted under greenhouse conditions and the silencing frequency was found to increase between experiment 1 (performed in mid-winter) and experiment 3 (performed in spring), suggesting that optimisation of growth conditions could further increase silencing efficiencies. In barley, efficiency of BSMV-mediated VIGS is highly cultivar dependent, and screening of different *B. distachyon *accessions may also further improve the VIGS efficiency in this species. For the experiments reported here, line Bd21-3 was chosen since this line is also the preferred line for transformation [18] and for mutagenesis and TILLING projects by international consortia (e.g., http://www.renewall.eu/). In a recent publication [7], successful VIGS was demonstrated in the *B. distachyon *ecotype ABR-1.

Our attempts at using BSMV for VIGS in oat were less promising. Although BSMV^CV42 ^and pseudorecombinants including BSMV^CV42^-α accumulated to similar levels in infected oat plants as in barley (Figure 6), we found it more difficult to achieve high infection frequencies with *in vitro *transcripts in oat compared to barley and *B. distachyon*, and the infected plants showed no or very limited signs of silencing. Although we screened a range of both hexaploid and diploid oat accessions for BSMV susceptibility before choosing two lines for the silencing experiments, it is possible that other accessions may be more suited for VIGS. Furthermore, it is known that growth conditions can significantly affect VIGS efficiency [42]. The experiments reported here were performed in growth chambers under conditions that were previously found to be appropriate for BSMV-mediated VIGS in barley [28], but further optimisation of temperature and light regimes might lead to improved silencing in oat.

Although the BSMV vectors are generally easy to manipulate during cloning work, occasional cloning difficulties prompted us to explore two previously reported systems for ligation-free cloning. Both the USER cloning strategy [40] and the T4 DNA polymerase strategy [41,53] rely on the generation of relatively short sticky ends for inserting fragments into the vectors; in the BSMV vectors reported here, only 8 nt are used for annealing. This means that in contrast to e.g. the GATEWAY cloning strategy, only very few extra nucleotides are introduced into the final virus construct relative to traditional restriction enzyme based cloning strategies. This is particularly important for vectors such as BSMV, where instability of the virus construct increases significantly with increasing length of the foreign sequence [28]. We found the USER strategy to be extremely efficient, fast and reliable for inserting both simple and more complex, inverted repeat fragments into the BSMV vector. The T4 DNA polymerase strategy as implemented here was slightly less efficient, with 6/12 clones containing the correct insert (8/12 in a subsequent experiment (data not shown)). However, due to the general availability of T4 DNA polymerase in most molecular biology laboratories some laboratories may find the T4 vector system preferable.

## Conclusions

In conclusion, we have shown that BSMV can be used to induce gene silencing in barley roots. However, we recommend the screening of potential BSMV constructs for stability prior to initiating root silencing experiments. Furthermore, BSMV was shown to be a promising tool for functional genomics studies in *B. distachyon*, but less so in oat. Finally, new versions of the BSMV vector adapted for ligation-free cloning enable fast and highly efficient insertion of simple as well as inverted-repeat silencing inducing fragments.

## Methods

### Infectious clones

Infectious clones of BSMV strain ND18 were from Prof. Andrew Jackson, University of Berkeley [2]. Modifications of the γ clone to allow insertion of gene fragments (BSMVγ-MCS) have previously been described [28]. These ND18-based clones are referred to as "BSMV^ND18^" or simply as "BSMV" in this paper. An ND18-based γ clone where expression of the γb open reading frame is blocked by an insertion site for gene fragments (termed BSMV-γΔb), as well as infectious clones of BSMV strain CV42, were obtained from Prof. Michael Edwards, USDA ARS, USA. The CV42-based clones are referred to here as "BSMV^CV42^".

### Plant growth conditions

Experiments with *B. distachyon *used the diploid inbred line Bd21-3 [18] and were carried out in a greenhouse with a 20/18°C day/night temperature regime and a light period of 16 h/day, regulated with supplementary light from September to May. Plants were grown in 6-cm peat pots with Pindstrup no. 2 Peat Substrate. Experiments with oat and barley were performed in growth chambers with constant temperature (20°C) and a 16 h light period with light intensity 300 μmol · m^-2 ^· s^-1 ^photosynthetic active radiation (PAR). Oat plants were sown in 11-cm pots with garden soil. For hydroponic culture of barley, seeds were germinated in vermiculite soaked with the final nutrient solution for 5 days (with a 24/17°C day/night temperature regime and 16 h light/day), then transferred to hydroponics with four plants per container. All plants (showing virus symptoms) in one container were treated as one sample. Each container contained one liter of aerated nutrient solution with the following composition: 0.2 mM K_2_SO_4_, 0.3 mM MgSO_4_, 0.1 mM NaCl, 0.3 mM Mg(NO_3_)_2_, 0.9 mM Ca(NO_3_)_2_, 0.6 mM KNO_3_, 0.05 mM Fe(III)-EDTA-Na, 0.5 μM MnCl_2_, 0.7 μM ZnCl_2_, 0.8 μM CuSO_4_, 2 μM H_3_BO_3_, 0.8 μM Na_2_MoO_4_. KH_2_PO_4 _was added separately to some containers to 1 mM final concentration. The pH was between 5.5 and 6.5. Nitrates and micronutrients were supplemented every day and the rest of the nutrients every 4 days. The Pi content in hydroponics was monitored every 4 days by inorganic Pi assay [54] and when necessary replenished. For growth of barley in soil, seeds were germinated on moist filter paper for 3 days in the growth chamber, then transferred to 11-cm pots with garden soil. In the experiments shown in Figure 3 each pot contained three plants that were treated as one sample. In the experiment shown in Figure 4 each pot contained one plant.

### RNA isolation

Total RNA was isolated from plant leaves or roots by use of the RNeasy Plant Mini kit (Qiagen, Valencia, CA, USA) following the manufacturers protocol with RLT buffer without β-mercaptoethanol and on-column DNase treatment.

### Construction of BSMV-USER and BSMV-T4

The BSMV-MCS vector has been described previously [28]. To create pBSMVγ-USER, two 5'-phosphorylated DNA oligos GCTGAGGCTTAATTAAACCTCAGC and GATCGCTGAGGTTTAATTAAGCCTCAGCAT were annealed and ligated into *Pac*I/*Bam*HI digested pBSMVγ-MCS. To prepare the vector for insertion of PCR products the plasmid was linearized with *Pac*I, ethanol precipitated and redissolved, digested with Nt.BbvCI, phenol-chloroform extracted, and redissolved at 0.02 pmol/μl. The prepared vector can be stored in aliquots at -20°C for at least one year. To create pBSMVγ-T4, two 5'-phosphorylated DNA oligos CGGATATTAATTAATTCCC and GATCGGGAGTTAATAAATATCCGAT were annealed and ligated into *Pac*I/*Bam*HI digested pBSMVγ-MCS. To prepare the vector for insertion of PCR products the plasmid was linearized with *Pac*I, followed by treatment with T4 DNA polymerase in the presence of 1 mM dCTP for 30 min. at 22°C. After heat-inactivation at 75°C for 20 min. the vector concentration was adjusted to 0.02 pmol/μl and stored at -20°C.

### VIGS constructs

cDNA was synthesized from 1 μg of total RNA using oligo(dT)_15 _primer (Promega, Madison, WI, USA) and M-MLV reverse transcriptase (Invitrogen, Carlsbad, CA, USA). Gene fragments for insertion into BSMV were PCR amplified using Expand High Fidelity PCR System (Roche Diagnostics, Mannheim, Germany) with the primers shown in Additional file 7. GenBank accession numbers for barley genes are also shown in Additional file 7. GFP fragments were amplified using pBIN-mgfp5-ER as template (GenBank Acc. No. U87974[55]). For insertion into pBSMV-MCS, PCR products were cloned into the pCR2.1-TOPO plasmid (Invitrogen), verified by sequencing, then excised by *Pac*I/*Bam*HI digestion and ligated into pBSMVγ-MCS. For insertion into pBSMVγ-USER, 0.2 pmol PCR product was mixed with 1 μl prepared vector (see above), 1 μl USER enzyme (New England Biolabs, Ipswich, MA, USA), and 1xPCR buffer to a total of 10 μl, incubated at 37°C for 20 min. followed by 20 min. at 25°C, then used directly for transformation of chemically competent *E. coli *cells. For insertion into pBSMVγ-T4, PCR products were purified on GFX columns, then treated with T4 DNA polymerase in the presence of 1 mM dGTP for 30 min. at 22°C. After heat-inactivation at 75°C for 20 min, 0.4 pmol PCR product was mixed with 1 μl prepared BSMV-T4 vector, incubated 5 min. at 22°C and used for transformation of *E. coli*.

The inverted repeat of *HvCel1 *was assembled from two PCR amplified fragments made with primers HvCel1 Fwd1+ HvCel1 Rev1, and HvCel1 Fwd2 + HvCel1 Rev2 (Additional file 7). The two PCR products were simultaneously inserted into pBSMVγ-USER as described above. This generates a 2 × 58 bp inverted repeat derived from the *HvCel1 *sequence with an 8 nt loop.

All fragments inserted into BSMV were from coding regions of the respective genes except for *HvIPS1*, which does not encode a protein.

### Plant inoculation

The procedures for *in vitro *transcription and inoculation have been described previously [28]. Plants were inoculated on the first leaf either six or seven days after sowing. For each experiment all plants were inoculated at the same age. All samples for analysis were harvested nine days after inoculation unless stated otherwise. Only plants displaying virus symptoms on their leaves at the time of harvesting were included in the analysis.

### Viral construct stability analysis by PCR

Touchdown PCR with Expand High Fidelity PCR System (Roche) was used to estimate BSMVγ construct stability with the following primers: BSMVgbF 5'-GAAGAAGATGCAGGAGCTGAA-3'; BSMVgbR 5'-CACTCCCATCATATGGTTGAT-3', these primers surround the inserted fragment (see Figure 1B).

Touchdown PCR was performed as previously described with one modification - elongation at 72°C - 1 min [56].

### Real-time qRT-PCR analysis

cDNA was synthesized from 200 ng of total RNA using random hexamer primers (Eurofins MWG Operon, Ebersberg, Germany) and Expand Reverse Transcriptase (Roche).

Real-time PCR analysis was performed on an Applied Biosystems 7500 system using SYBR Green Master Mix (Applied Biosystems, Foster City, CA, USA) and the Absolute Quantification method according to the manufacturer's protocol. Data were normalized to ubiquitin and/or 18 S rRNA levels. Primers used for real-time PCR are shown in Additional file 7. Real Time PCR products for *HvPht1;1*, *HvIPS1*, *HvPHR1*, and ubiquitin have been verified by sequencing. After each PCR reaction, the specificity of the amplification was verified by melting curve analysis. All samples were measured in duplicate.

### Inorganic Pi measurements

The content of inorganic Pi in plant root samples were determined as previously described [57].

### DAS-ELISA

The standard protocol for DAS-ELISA [58] was used with antisera against BSMV (Cat. No. AS-0135) from DSMZ GmbH, Braunschweig, Germany.

## Competing interests

The authors declare that they have no competing interests.

## Authors' contributions

AP carried out most of the work on VIGS in barley roots, drafted part of the manuscript and produced figures. KG was responsible for the work on VIGS in oat, drafted part of the manuscript and produced figures. BJ performed the work on VIGS in *B. distachyon*. MB-P was responsible for the hydroponics experiments and performed experiments involving *HvIPS1*. LN participated in the bioinformatics work and supervised the set-up of the hydroponics system. MG participated in the VIGS experiments in barley roots. THN, EJ, IJ and MA participated in the design and coordination of the study. MA contributed to the molecular work and finalised the manuscript. All authors contributed to and approved the final manuscript.

## Supplementary Material

Additional file 1 **Pi content in hydroponics: HvPht1;1 experiment. Format: PDF**. Pi content in the roots of plants inoculated with either BSMV-Pht1;1 (black bars) or BSMV-GFP^375 ^(white bars) shown as μmol/g of fresh weight. Plants were grown in hydroponic cultures with 0 or 1 mM Pi and harvested 9 days after inoculation. Data from experiment shown in Figure 1. Error bars indicate standard deviations. Click here for file

Additional file 2 **Alignment of *PDS *sequences. Format: WORD**. Nucleotide sequence alignment of partial *PDS *sequences from *B. distachyon *(BdPDS: HM755884) and *A. strigosa *(AsPDS; HM755676) with other monocot *PDS *sequences. Barley sequence (HvPDS): AY062039. Wheat (TaPDS): FJ517553. Maize (ZmPDS): L39266. Rice (OsPDS): AF049356. Nucleotides conserved in all six sequences are shaded black. Dark grey shaded nucleotides are conserved in five out of six sequences, and light grey in four out of six. Click here for file

Additional file 3 **BSMV in *B. distachyon *leaves and roots. Format: PDF**. BSMV coat protein detection by DAS-ELISA in leaves and roots of *B. distachyon *plants infected with BSMV-MCS. Click here for file

Additional file 4 **Lack of silencing of *AsCel1 *in oat shoots. Format: PDF**. *AsCel1 *RNA expression levels determined by qRT-PCR (normalization to 18 S rRNA). *A. strigosa *cv. S75 plants were inoculated with either BSMV-GFP^375^, BSMV-AsCel1-2 or BSMV-AsCel1-3. From left to right, the bars represent 5, 6 and 6 samples, respectively. Error bars denote standard deviations. AU - Arbitrary units. Differences between averages are not significant (*p *> 0.1, Student's t-test). Click here for file

Additional file 5 **GC content in silencing fragments. Format: PDF**. Percentage GC content in fragments inserted into the BSMV vector. Click here for file

Additional file 6 **Pi content in hydroponics: HvIPS1 experiment. Format: PDF**. Pi content in the roots of plants inoculated with either BSMV-IPS1 (black bars) or BSMV-GFP^250 ^(white bars) shown as μmol/g of fresh weight. Plants were grown in hydroponic cultures with 0 or 1 mM Pi and harvested either at 5 dpi, 7 dpi, or 9 dpi. Data from experiment shown in Figure 2A. Error bars indicate standard deviations. Click here for file

Additional file 7 **Primers used and barley gene GenBank accession numbers. Format: WORD**. Click here for file
