# Supplementary material for: Investigations of barley stripe mosaic virus as a gene silencing vector in barley roots and in Brachypodium distachyon and oat
Source: Plant Methods. 2010 Nov 30;6:26. doi: 10.1186/1746-4811-6-26 (PMC3006357; doi:10.1186/1746-4811-6-26)
Supplement: Additional file 1 — Pi content in hydroponics: HvPht1;1 experiment. Format: PDF. Pi content in the roots of plants inoculated with either BSMV-Pht1;1 (black bars) or BSMV-GFP375 (white bars) shown as μmol/g of fresh weight. Plants were grown in hydroponic cultures with 0 or 1 mM Pi and harvested 9 days after inoculation. Data from experiment shown in Figure 1. Error bars indicate standard deviations. [file 1746-4811-6-26-S1.PDF]

Additional file 1: Pi content in hydroponics: HvPht1;1 experiment

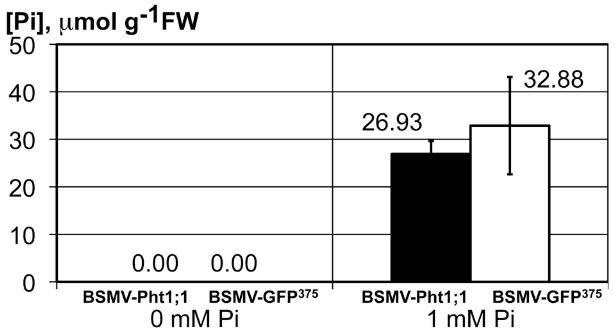

Pi content in the roots of plants inoculated with either BSMV-Pht1;1 (black bars) or BSMV-GFP[375] (white bars) shown as µmol /g of fresh weight. Plants were grown in hydroponic cultures with 0 or 1 mM Pi and harvested 9 days after inoculation. Data from experiment shown in Fig. 1. Error bars indicate standard deviations.
