# Supplementary material for: Investigations of barley stripe mosaic virus as a gene silencing vector in barley roots and in Brachypodium distachyon and oat
Source: Plant Methods. 2010 Nov 30;6:26. doi: 10.1186/1746-4811-6-26 (PMC3006357; doi:10.1186/1746-4811-6-26)
Supplement: Additional file 2 — Alignment of PDS sequences. Format: WORD. Nucleotide sequence alignment of partial PDS sequences from B. distachyon (BdPDS: HM755884) and A. strigosa (AsPDS; HM755676) with other monocot PDS sequences. Barley sequence (HvPDS): AY062039. Wheat (TaPDS): FJ517553. Maize (ZmPDS): L39266. Rice (OsPDS): AF049356. Nucleotides conserved in all six sequences are shaded black. Dark grey shaded nucleotides are conserved in five out of six sequences, and light grey in four out of six. [file 1746-4811-6-26-S2.DOC]

Additional file 2: Alignment of *PDS* sequences.


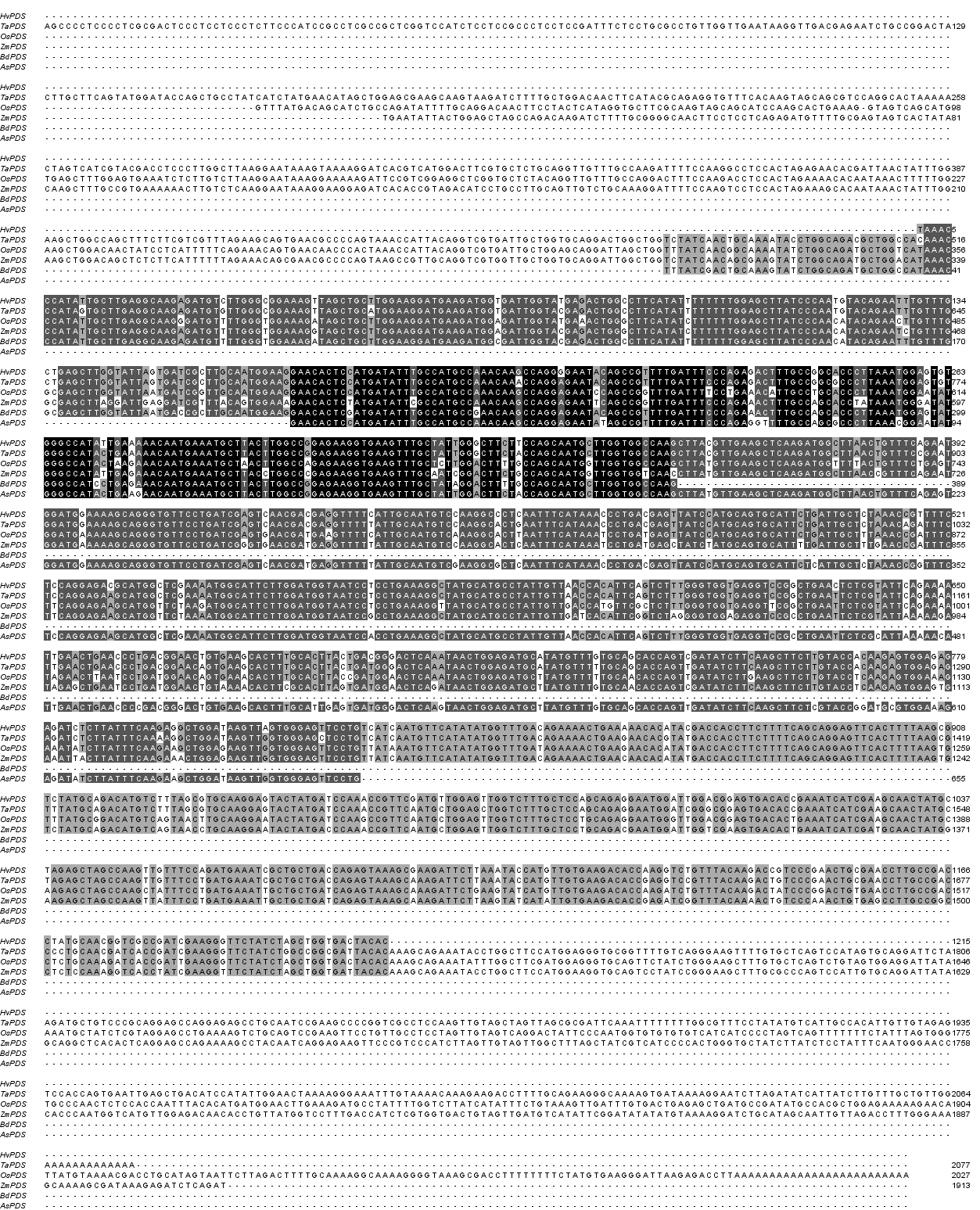


Nucleotide sequence alignment of partial *PDS* sequences from *B. distachyon* (BdPDS: HM755884) and *A. strigosa* (AsPDS; HM755676) with other monocot *PDS* sequences. Barley sequence (HvPDS): AY062039. Wheat (TaPDS): FJ517553. Maize (ZmPDS): L39266. Rice (OsPDS): AF049356. Nucleotides conserved in all six sequences are shaded black. Dark grey shaded nucleotides are conserved in five out of six sequences, and light grey in four out of six
