# Supplementary material for: Investigations of barley stripe mosaic virus as a gene silencing vector in barley roots and in Brachypodium distachyon and oat
Source: Plant Methods. 2010 Nov 30;6:26. doi: 10.1186/1746-4811-6-26 (PMC3006357; doi:10.1186/1746-4811-6-26)
Supplement: Additional file 3 — BSMV in B. distachyon leaves and roots. Format: PDF. BSMV coat protein detection by DAS-ELISA in leaves and roots of B. distachyon plants infected with BSMV-MCS. [file 1746-4811-6-26-S3.PDF]

Additional file 3: BSMV in *B. distachyon* leaves and roots

BSMV coat protein detection by DAS-ELISA in leaves and roots of *B. distachyon* plants infected with BSMV-MCS.

	**OD 405 nm**	
**Sample**	**Leaves**	**Root**
Healthy plant 1	0.098	0.027
Healthy plant 2	0.012	0.012
BSMV-MCS plant 1	1.573	1.322
BSMV-MCS plant 2	1.602	1.188
BSMV-MCS plant 3	1.734	1.062
BSMV-MCS plant 4	1.487	0.990
BSMV-MCS plant 5	1.384	0.786
BSMV-MCS plant 6	1.506	1.370
BSMV-MCS plant 7	1.670	1.026
BSMV-MCS plant 8	1.391	1.018
BSMV-MCS plant 9	1.385	1.231
BSMV-MCS plant 10	1.444	0.867
BSMV-MCS plant 11	1.578	0.712
BSMV-MCS plant 12	1.444	1.089
Buffer	0.020	
