# Supplementary material for: Investigations of barley stripe mosaic virus as a gene silencing vector in barley roots and in Brachypodium distachyon and oat
Source: Plant Methods. 2010 Nov 30;6:26. doi: 10.1186/1746-4811-6-26 (PMC3006357; doi:10.1186/1746-4811-6-26)
Supplement: Additional file 4 — Lack of silencing of AsCel1 in oat shoots. Format: PDF. AsCel1 RNA expression levels determined by qRT-PCR (normalization to 18 S rRNA). A. strigosa cv. S75 plants were inoculated with either BSMV-GFP375, BSMV-AsCel1-2 or BSMV-AsCel1-3. From left to right, the bars represent 5, 6 and 6 samples, respectively. Error bars denote standard deviations. AU - Arbitrary units. Differences between averages are not significant (p > 0.1, Student's t-test). [file 1746-4811-6-26-S4.PDF]

Additional file 4: Lack of silencing of *AsCel1* in oat shoots

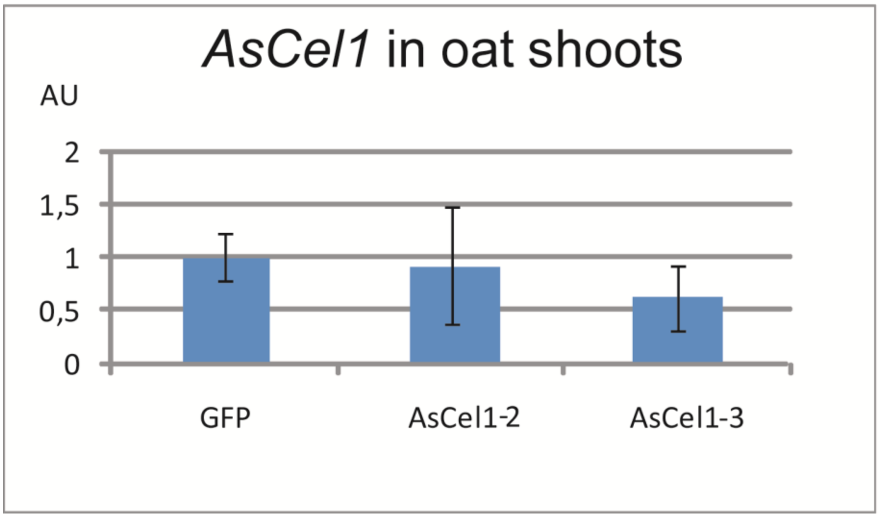

AsCel1 RNA expression levels determined by qRT-PCR (normalization to 18S rRNA). *A. strigosa* cv. S75 plants were inoculated with either BSMV-GFP^{375}, BSMV-AsCel1-2 or BSMV-AsCel1-3. From left to right, the bars represent 5, 6 and 6 samples, respectively. Error bars denote standard deviations. AU – Arbitrary units. Differences between averages are not significant ($p > 0.1$, Student's t-test).
