# Supplementary material for: Investigations of barley stripe mosaic virus as a gene silencing vector in barley roots and in Brachypodium distachyon and oat
Source: Plant Methods. 2010 Nov 30;6:26. doi: 10.1186/1746-4811-6-26 (PMC3006357; doi:10.1186/1746-4811-6-26)
Supplement: Additional file 5 — GC content in silencing fragments. Format: PDF. Percentage GC content in fragments inserted into the BSMV vector. [file 1746-4811-6-26-S5.PDF]

Barley stripe mosaic virus as a gene silencing vector in barley roots and in *Brachypodium distachyon* and oat

Additional file 5: GC content in silencing fragments

Percentage GC content in fragments inserted into the BSMV vector.

Gene fragment	GC%
HvPht1;1	58.1
HvIPS1	41.8
HvPHR1	47.4
$HvPHO2^{247}$	46.2
HvCel1-1	46.6
HvCel1-3	54.4
HvCel1-IR	53.4
