# Supplementary material for: Investigations of barley stripe mosaic virus as a gene silencing vector in barley roots and in Brachypodium distachyon and oat
Source: Plant Methods. 2010 Nov 30;6:26. doi: 10.1186/1746-4811-6-26 (PMC3006357; doi:10.1186/1746-4811-6-26)
Supplement: Additional file 6 — Pi content in hydroponics: HvIPS1 experiment. Format: PDF. Pi content in the roots of plants inoculated with either BSMV-IPS1 (black bars) or BSMV-GFP250 (white bars) shown as μmol/g of fresh weight. Plants were grown in hydroponic cultures with 0 or 1 mM Pi and harvested either at 5 dpi, 7 dpi, or 9 dpi. Data from experiment shown in Figure 2A. Error bars indicate standard deviations. [file 1746-4811-6-26-S6.PDF]

Additional file 6: Phosphate content in hydroponics: *HvIPS1* experiment

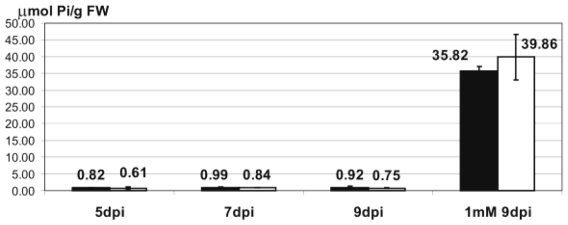

Phosphate content in the roots of plants inoculated either with BSMV-IPS1 (black bars) or BSMV-GFP^{250} (white bars). Plants were grown in hydroponic culture with 0 or 1 mM phosphate and harvested either 5 days postinoculation (dpi), 7dpi, or 9dpi. Data from experiment shown in Figure 2a.
