# Supplementary material for: Investigations of barley stripe mosaic virus as a gene silencing vector in barley roots and in Brachypodium distachyon and oat
Source: Plant Methods. 2010 Nov 30;6:26. doi: 10.1186/1746-4811-6-26 (PMC3006357; doi:10.1186/1746-4811-6-26)
Supplement: Additional file 7 — Primers used and barley gene GenBank accession numbers. Format: WORD. [file 1746-4811-6-26-S7.DOCX]

**Additional file 7: Primers used and barley gene GenBank accession numbers**

| **Primers used for fragments to be inserted into BSMV** | | | |
| --- | --- | --- | --- |
| HvPht1;1 | | Fwd 5’-ATTTTAATTAAATTTGGTACTATTGTTACCATC-3’  Rev 5’-ATTGGATCCTTGTGGTCGCTAGCAAGTG-3’ | 368 bp |
| HvIPS1 | | Fwd 5’-TTAATTAAGTAACTTCTCGACCGGG-3’  Rev 5’ GGATCCTAACAAAAGATCATATTACC-3’ | 251 bp |
| HvPHR1 | | Fwd 5’-TTAATTAAGACTACAGGGATAACATCC-3’  Rev 5’-GGATCCTAGCTACATTGCAAATCTCTC-3’ | 253 bp |
| HvPht1;4 | | Fwd 5’-ATTTTAATTAAGCCGGCGGCATCGTCAC-3’  Rev 5’-ATTGGATCCGCCGTGCCGACGAGGTG-3’ | 374 bp |
| HvPht1;7 | | Fwd 5’-ATTTTAATTAAGCGTGGCGATCGGGATC-3’  Rev 5’-ATTGGATCCTCGACGCCGTGCCCAAG-3’ | 381 bp |
| HvPHO2^247^ | | Fwd 5’-GGCTTAAUGGTGATTGGTGCACTCTC-3’  Rev 5’-GGTTTAAUCGGCAGCTTCCTCTACTG-3’ | 247 bp |
| HvPHO2^387^ | | Fwd 5’-GGCTTAAUGGTGATTGGTGCACTCTC-3’  Rev 5’-GGTTTAAUTAGCTCATAAGCACTCACTG-3’ | 387 bp |
| HvCel1-1 | | Fwd 5’-CCTTAATTAAcattCTAGGCAAGAACCCAC-3’  Rev 5’-ACGGATCCttcaaggtttccaagctga-3’ | 401 bp |
| HvCel1-3 | | Fwd 5’-CCTTAATTAAggacagcaaggcgtactct-3’  Rev 5’-ACGGATCCtatgagcacatgacattgtc-3’ | 401 bp |
| HvCel1-IR | | Fwd1 5’-GGCTTAAUGGGATGACAAGCTTCCAGG-3’  Rev1 5’-ATAATGGGCUTAGGAAGAGCCGCAACCTG-3’  Fwd2 5’-AGCCCATTAUCTTAGGAAGAGCCGCAAC-3’  Rev2 5’-GGTTTAAUGGGATGACAAGCTTCCAG-3’ | 58 bp IR  8 nt loop |
| GFP^250^ | | Fwd 5’-TTAATTAAgacaccctcgtcaacagg-3’  Rev 5’-GCGGATCCAATGGTTGTCTGGTAAAAG-3’ | 250 bp |
| GFP^375^ | | Fwd 5’-TTAATTAACAGATCATATGAAGCGGCAC-3’  Rev 5’- gcggatccaatggttgtctggtaaaag-3’ | 375 bp |
| BdPDS-USER | | Fwd 5’-GGCTTAAUCCAAGCATTGCTGGCAAAAG-3’  Rev 5’-GGTTTAAUAGCTGCTTGGAAGGATGAAG-3’ | 300 bp |
| BdPDS-T4 | | Fwd 5’-GGATATTACCCAAGCATTGCTGGCAAAAG-3’  Rev 5’-GGGAATTACAGCTGCTTGGAAGGATGAAG-3’ | 300 bp |
| AsPDS1 | | Fwd 5- CCTTAATTAAGCAGGGTGTTCCTGATCGAGT-3’  Rev 5’- TGGGATCCCAGGAACTCCCACCAACTTATC-3’ | 422 bp |
| AsPDS2 | | Fwd 5’- CGTTAATTAAGAACACTCCATGATATTTGCCATGC-3’  Rev 5’- TGGGATCCGGAGGATTACCATCCAAGAATGC-3’ | 401 bp |
| AsCel1-1 | | Fwd 5’- CCTTAATTAACATTCTAGGCAAGAACCCAC-3’  Rev 5’- ACGGATCCTTCATGGTTTCCAAGCTGA-3’ | 404 bp |
| AsCel1-2 | | Fwd 5’- CCTTAATTAAGACATTCCACAACCAAACTGAC-3’  Rev 5’- ACGGATCCGACTCCCTCCATTTGA-3’ | 399 bp |
| AsCel1-3 | | Fwd 5’- CCTTAATTAAGGACAGCAAGGCGTACTCT-3’  Rev 5’- ACGGATCCTATGAGCACATGACATTGTC-3’ | 399 bp |
| **Primers used for real-time PCR:** | | | |
| HvPht1;1 | Fwd 5’-GACACAGAGTCATGCACAAG-3’  Rev 5’- TAAGCGACACCACAAATTCAG-3’ | | 89 bp |
| HvIPS1 | Fwd 5’-CACGCTCCGTTATCCAATCC-3’  Rev 5’-CACACTCCGAGTGAGGATG-3’ | | 123 bp |
| HvPHR1 | Fwd 5’-TCTGCCAAATTCTCTGAAAG-3’  Rev 5’-CAAAGTAGCAGACGGAAATG-3’ | | 124 bp |
| HvPHO2 | Fwd 5'-GACGAGAGCATCGGCAAG-3'  Rev 5'-CACCAGAGTATGTCTCCAAG-3' | | 151 bp |
| HvCel1 | Fwd 5’-GTGGGTGACACCTCTAAAGG-3’  Rev 5’-AAGATCCGAGCAGGAGTGG-3’ | | 111 bp |
| BdPDS | Fwd 5’-CTTTCCACCCAAAACATCTCTT-3’  Rev 5’-GGTTTATCGACTGCAAAGTATC-3’ | | 81 bp |
| AsCel1 | Fwd 5’-ACTCTTGCAGCAAATGCTGG-3’  Rev 5’-GAACAGCTGAGAAGATGGTG-3’ | | 106 bp |
| Ubiquitin | Fwd 5’-GCGTGGTGGCAAGTAAGTG-3’  Rev 5’-AATGGAAACAACGACACAACC-3’ | | 120 bp |
| 18S rRNA | Fwd 5’-GACTACGTCCCTGCCCTTTG-3’  Rev 5’-AACACTTCACCGGACCATTCA-3’ | | 68 bp |

The lengths of the fragments shown are without restriction sites or USER recognition sites

Primers for phosphate transporters, *HvCel1*, ubiquitin and 18S rRNA were designed based on existing GenBank data: *HvPht1;1* – AF543197, *HvPht1;4* – AY187024, *HvPht1;7* – AY187022, *HvCel1* – AB040769, ubiquitin – X04133, 18S rRNA – M82668.

Sequences for the putative barley homologues of Arabidopsis *AtPHR1*, *AtIPS1* and *AtPHO2* have been submitted to GenBank: *HvPHR1* – GQ337895 *HvIPS1* – GQ301528, *HvPHO2* – GQ861514.
